# Rare Disorders: Diagnosis and Therapeutic Planning for Patients Seeking Orthodontic Treatment

**DOI:** 10.3390/jcm11061527

**Published:** 2022-03-10

**Authors:** Carolina Arriagada-Vargas, María Teresa Abeleira-Pazos, Mercedes Outumuro-Rial, Eliane García-Mato, Iván Varela-Aneiros, Jacobo Limeres-Posse, Pedro Diz-Dios, Márcio Diniz-Freitas

**Affiliations:** Medical-Surgical Dentistry Research Group (OMEQUI), Health Research Institute of Santiago de Compostela (IDIS), University of Santiago de Compostela (USC), 15782 Santiago de Compostela, Spain; caarriagadav@gmail.com (C.A.-V.); maite.abeleira@usc.es (M.T.A.-P.); mercedesoutumuro@hotmail.com (M.O.-R.); eliane.garma@gmail.com (E.G.-M.); ivan.varela.aneiros@gmail.com (I.V.-A.); pedro.diz@usc.es (P.D.-D.)

**Keywords:** rare diseases, orthodontics, dysgnathia, malocclusion, orofacial manifestations, dental care

## Abstract

The available literature on the orthodontic treatment of patients with rare disorders is extremely scarce. The aim of this study was to analyze the diagnosis and orthodontic treatment of a group of 94 individuals with rare diseases, referred for orthodontic evaluation to a university special care dentistry center (University of Santiago de Compostela, Spain). We created a control group of 94 systemically healthy individuals, paired by sex and age range. For all participants, we recorded their dental and skeletal abnormalities, oromotor dysfunctions and the characteristics of their orthodontic treatment. Some of the morphological and functional abnormalities were more prevalent in the rare disorders group than in the control group, including dental agenesis, microdontia, enamel defects, maxillary hypoplasia, overbite, cleft lip/palate, mouth breathing, atypical swallowing, lingual/labial interposition, labial incompetence, modified consistency diet, bruxism, and muscle tone abnormalities. Compared with the control group, the 56 patients with rare disorders who underwent orthodontic treatment required more desensitization sessions, used mixed appliances (fixed and removable) more often and for longer periods and had more frequent complications, such as gingivitis, caries, mucosal ulcers and recurrent debonding of the device. In conclusion, for selected patients with rare disorders, it is feasible to perform orthodontic treatment, whose planning will be determined by the dental-skeletal abnormalities and oromotor dysfunctions. Although complications are more frequent, they can typically be solved without having to stop treatment.

## 1. Introduction

According to the definition provided by the European Union, a disease is classified as “rare” when its prevalence is less than 1 in every 2000 individuals [1]. More than 6000 rare diseases (RD) have been reported, which, in Europe alone, affect more than 30 million individuals [2]. Eighty percent of RDs are genetic in origin, have considerable phenotypic variability, and are often chronic and/or degenerative in nature. In 65% of cases, their prognosis is severe [3]. Common characteristics of RDs include the difficulty in reaching a definitive diagnosis (which generally is delayed or even unreachable), the current lack of scientific knowledge on many of these diseases, the unequal access to health care, the resulting societal consequences, and the high cost of certain treatments [2,4].

In a considerable percentage of RDs of genetic origin, some of the genes involved can have the same signaling pathways that regulate certain orofacial structures; the clinical manifestations that affect this anatomical region are, therefore, common [5]. In the framework of a program started in Germany for developing a database to record the orofacial manifestations of patients with RDs (ROMSE, Porta Westfalica, Germany), at least 471 RDs were identified with various types of dental abnormalities, mouth lesions, dental-skeletal abnormalities, and cleft lip/palate [6].

It has been suggested that the most common orofacial abnormalities correspond to abnormalities in the formation and number of teeth, dysgnathia and malocclusions [7]. In addition to orofacial dysmorphology, more than 40% of individuals with RDs have oromotor dysfunction [8,9]. All of these conditions negatively affect the oral health-related quality of life [7], especially from the psychosocial standpoint, in terms of the course of treatment and access to dental care [10].

Studies have indicated that dentists should maintain a close professional relationship with healthcare practitioners in multidisciplinary teams for the care of patients with RDs (e.g., geneticists), because the latter practitioners can be the first to suspect that a patient has a previously undiagnosed genetic disorder or, if the patient is already diagnosed with an RD, can access essential information on how the patient’s systemic condition can affect their dental treatment [11]. In recent decades, there has been growing interest in the relationship between RDs and odontology, which has been expressed in the development of research networks and specific databases and in the establishment of reference centers for dental care [12,13]. However, individuals with RDs still have less access to oral health care than the general population [14,15], and most dentists (except for those who work in university hospitals) have little or no knowledge of RDs [16].

Almost 1 of every 3 identified RDs have a relevant orthodontic manifestation (e.g., dysgnathia), and there is information on the orthodontic diagnosis from specific RD databases [17] and from highly important series such as the Mun-H-Center (https://www.mun-h-center.se, accessed on 12 December 2021), a Swedish national orofacial resource center for RD [8]. However, the literature available on the orthodontic treatment of these patients is extremely scarce and is limited to single case studies or small series of a specific RD [18,19]. A notable exception is the series reported by Sjögreen et al. [8], which included 1829 individuals, mostly from the Ågrenska National Competence Centre for Rare Diseases (Gothenburg, Sweden), a considerable percentage of whom underwent orthodontic treatment, although the authors provided no information on the characteristics of the procedures performed.

The main objective of our study was to provide a general overview of the diagnosis and orthodontic treatment of patients with RDs, based on our experience in a university center of reference for special care dentistry.

## 2. Materials and Methods

This was a descriptive, retrospective, longitudinal study, designed by applying a classical case-control regimen. The participants (or their legal guardians, if necessary) signed an informed consent authorizing the use of the information contained in their medical history. The research study and the use of this information with purposes of disclosure were approved by the Ethics Committee of the University of Santiago de Compostela (Spain).

### 2.1. Selection of Study and Control Groups

The study group or RD group (RDG) consisted of 94 individuals (45 male and 49 female participants; age range 3–50 years; median age, 16 years) with an RD, who had undergone care in the Odontology Unit for Patients with Special Needs of the University of Santiago de Compostela (UOPNE-USC, Spain). This was a convenience study group, selected by applying the following inclusion criteria: having a confirmed diagnosis of an RD with systemic repercussion (https://www.orpha.net/consor/cgi-bin/index.php, accessed on 12 December 2021); having attended an orthodontic consultation in UOPNE-USC between 2002 and 2020; and having a complete medical history, including a confirmed systemic diagnosis and orthodontic dental diagnosis (Supplementary Materials Figures S1–S4).

Moreover, the individuals in the RDG were distributed into 8 categories, taking into account the main target organ/system affected, namely, neurological disorders, global developmental disorders, skeletal dysplasia, head and neck syndromes, genodermatosis, sensory abnormalities, intellectual/cognitive impairment and miscellaneous (when the impairment was highly heterogeneous and impeded its categorization).

We created a systemically healthy control group (SHCG) of 94 individuals who underwent orthodontic treatment during the same period and by the same practitioners. The sample selection was stratified by type, because the SHCG patients were included in the study paired by sex and age range (≤13 years and >13 years) with respect to the RDG. The age range of the SHCG was 7–38 years (median, 17 years), 45 were female, and 49 were male.

### 2.2. Description of Variables

For all study participants, we recorded the variables related to oral manifestations, oral functionality, and orthodontic treatment.

The oral manifestations were analyzed dichotomously (presence/absence) and classified into 2 groups: dental and orthodontic. Within the dental characteristics, we studied 4 types of abnormalities: numerical, eruptive, morphological, and structural.

The orthodontic characteristics were analyzed in the 3 spatial planes: sagittal or anteroposterior, vertical, and transversal. In the anteroposterior direction, the maxillo–mandibular relationships were categorized according to Angle’s classification of malocclusion (classes I, II and III), based on the relationship between the first molars and the type of mandibular growth. In the vertical plane, we assessed the facial biotype (mesofacial, brachyfacial, dolichofacial) and the presence of open bite or overbite. In the transverse plane, the recorded abnormalities were the presence of crossbite, scissor bite and maxillary compression. We also verified the presence or not of cleft lip/palate.

The functional variables were generally determined based on the clinical findings, were assessed dichotomously (yes/no), and included the following: mouth breathing, labial incompetence, lingual interposition, atypical swallowing, diet (oral and consistency), bruxism and muscle tone abnormalities (hypotonia/hypertonia).

### 2.3. Statistical Analysis

The statistical analysis of the results was performed using the free software R [20]. For all participants in the RDG and SHCG, we performed a descriptive analysis (in terms of prevalence) of all the recorded variables regarding the oral manifestations, oral functionality, and orthodontic treatment. For the RDG, we also performed a descriptive analysis of all the recorded variables based on the 8 established categories, taking into account the main target organ/system affected.

To compare the results from the RDG and SHCG, we applied the chi-squared test of independence and Fisher’s exact test, performing a bivariate statistical analysis by association and correlation. If the *p*-value was greater than 0.05, the null hypothesis was rejected, and the variables were considered dependent. We also applied the Wilcoxon rank-sum test (when the samples did not follow a normal distribution or were too small to determine whether they actually came from normal populations) and the Kruskal–Wallis test (a non-parametric method for checking whether a group of data comes from the same population).

## 3. Results

### 3.1. Characterization of the Rare Disease Patient Group

The 94 patients of the RDG were distributed into the 8 pre-established categories according to the target organ/system as follows: 25 had neurological disorders, 12 had global developmental abnormalities, 10 had skeletal dysplasia, 10 had head and neck syndromes, 9 had genodermatosis, 4 had sensory disorders, 11 had intellectual/cognitive impairment, and 11 were included in the miscellaneous category (Table 1).

The most prevalent diagnoses in the RDG were ectodermal dysplasia (*n* = 7), fragile X syndrome (*n* = 4), periventricular leukomalacia (*n* = 3), microcephaly (*n* = 3), Williams syndrome (*n* = 3), Rett syndrome (*n* = 3), and Pierre Robin syndrome (*n* = 3). Exceptionally, we also included a number of patients with ultra rare diseases (fewer than 1 case in every 50,000 inhabitants), such as Myhre syndrome.

### 3.2. Anatomical Oral Manifestations in the Rare Disease Patient Group

Dental abnormalities were detected in 51 (54.2%) patients of the RDG. Tooth number anomalies were the most prevalent (27 patients; 28.7%), tooth eruption abnormalities were observed in 21 patients (22.3%), and morphology and dental structure abnormalities were observed in 14 (14.9%) and 12 patients (12.7%), respectively. With regard to each specific variable, agenesis was the most prevalent (23 patients; 24.4%), followed by ectopic eruption (18 patients; 19.1%) and enamel hypoplasia (11 patients; 11.7%). Taking into account the 8 categories in which the RDG patients were distributed, tooth agenesis was the most prevalent condition among the cases of genodermatosis (9 patients; 100%), and ectopic eruption and enamel hypoplasia were the most common among the head and neck syndromes (60% and 30%, respectively). These results are detailed in Table 2.

In terms of the intermaxillary relationship in the anteroposterior direction, Angle class III was the predominant pattern (39 patients; 41.4%), followed by class II (37 patients; 39.3%); only 18 patients (19.1%) presented normal occlusion. In the vertical direction, open bite was the most prevalent disorder and was detected in 26 patients (27.6%). In the transverse direction, the most common anomaly was maxillary compression, which was recorded in 41 patients (43.6%). Twenty patients (21.2%) were diagnosed with a bilateral crossbite. With regard to the facial biotype, the most prevalent was mesofacial (56 patients; 59.5%) followed by dolichofacial (34 patients; 36.2%). We confirmed the presence of a cleft lip/palate in 7 patients (7.4%). Taking into account the 8 categories into which the RDG patients were distributed, Angle class I was the predominant class in those diagnosed with genodermatosis (5 patients; 55.5%), class II was predominant in the neurological disorders category (15 patients; 60%) and in intellectual/cognitive impairment (6 patients; 54.5%), and class III was predominant among the patients with sensory disorders (3 patients; 75%), those with skeletal dysplasia (6 patients; 60%), those belonging to the miscellaneous category (7 patients; 53.8%), and those with global developmental abnormalities (6 patients; 50%). These results are detailed in Table 3.

In the vertical direction, open bite was particularly prevalent among the patients with sensory disorders (2 patients; 50%), in the miscellaneous category (6 patients; 46.2%), and in skeletal dysplasia (3 patients; 30%). Overbite was the most prevalent condition among the neurological disorders (6 patients; 24%). In the transverse direction, maxillary compression was especially frequent in the sensory disorders category (3 patients; 75%), in the head and neck syndromes (7 patients; 70%), and in the cases of skeletal dysplasia (6 patients; 60%). Bilateral crossbite was observed in 2 patients with sensory disorders (50%) and in 4 patients (40%) with head and neck syndromes. All of the patients with genodermatosis had a mesofacial biotype, as well as 81.8% of those in the intellectual/cognitive impairment category, 70% of those with skeletal dysplasia, and 70% of those with head and neck syndromes. A dolichofacial biotype was observed in 8 patients of the miscellaneous category (61.5%) and in 2 patients with sensory disorders (50%).

### 3.3. Oral Functionality in the Rare Disease Group

Functional abnormalities were detected in 74 (78.7%) RDG patients. The most common in decreasing order of frequency were mouth breathing (48 patients; 51%), labial incompetence (45 patients; 47.8%), lingual interposition (29 patients; 30.8%) and atypical swallowing (26 patients; 27.6%).

With regard to diet, most of the patients were fed orally (91 patients; 96.8%), and only 12 patients (13.1%) required a modification in diet consistency. Among the parafunctions, the most common was bruxism, which was detected in 25 patients (26.5%). Twenty-nine (30.8%) of the patients were hypotonic, while 22 patients (23.4%) had muscle hypertonia.

Taking into account the 8 categories in which the RDG patients were distributed, the highest rates of mouth breathing, atypical swallowing, lingual interposition, labial incompetence, and bruxism were detected in the head and neck syndrome group (80%), intellectual/cognitive disability group (45.4%), global developmental abnormality group (50%), miscellaneous group (76.9%), and the skeletal dysplasia group (40%), respectively. These results are detailed in Table 4.

### 3.4. Orthodontic Treatment in the Rare Diseases Group

Of the 94 individuals of the RDG who were orthodontically evaluated, 38 (40.4%) did not start treatment (Table 5). In 27 cases (71.0%), the decision was made by the practitioner, based mainly on the lack of tooth replacement (26 patients; 68.4%). In 26 cases (68.4%), the decision was made for reasons attributable to the patient, especially due to the lack of collaboration (21 patients; 55.2%), or by deficient oral hygiene (14 patients; 36.8%). Dental treatment was not started for only one patient (diagnosed with glycogen storage disease type I) because they had chronic mucositis, which was attributed to the administration of infliximab. In 22 cases (57.8%), the patients’ legal guardians did not agree to the starting of the orthodontic treatment.

In the neurological disorders category and global developmental disorders category, the most common causes for not starting the treatment were attributed to the patient (81.8% and 100%, respectively), essentially due to the lack of collaboration (81.8% and 83.3%, respectively). In the categories of skeletal dysplasia and intellectual/cognitive impairment, the decision was based primarily on the refusal by the legal guardians (75% and 83.3%, respectively). These results are detailed in Table 5.

Of the 56 patients (59.5% of the total) who started the treatment, 17 (30.3%) required a prior desensitization process. Fixed appliances (multibracket) were the most often used technique (49 patients; 87.5%). Forty-two patients (75%) showed some type of complication during the orthodontic treatment, especially clinical oral complications (31 patients; 55.3%) and, in particular, gingivitis (30 patients; 53.5%). Thirty-five patients (62.5%) presented some type of technical complication related to the appliance; the most common was recurrent debonding (29 patients; 51.7%). The start of the orthodontic treatment coincided in time with a worsening of the systemic condition in only 3 patients (5.3%). Complications required the temporary discontinuation of treatment in 12 patients (21.4%) and the definitive discontinuation in 4 cases (7.1%) (Table 6).

### 3.5. Comparison of the Results between the Rare Diseases Group and the Systemically Healthy Control Group

When comparing the prevalence of dental abnormalities in the RDG (*n* = 94) and the SHCG (*n* = 94), we observed statistically significant differences in favor of the RDG regarding the presence of agenesis (*p* < 0.001), microdontia (*p* = 0.027) and dental structure abnormalities, especially of the enamel (*p* = 0.010) (Table 7).

When analyzing the orthodontic variables (Table 8), the exclusive involvement of the maxilla was more frequent in the RDG than in the SHCG, while both bone bases (maxillo-mandibular) were more involved in the SHCG than in the RDG (*p* = 0.002). Overbite was more prevalent in the RDG than in the SHCG (*p* = 0.013). Cases of cleft lip/palate were detected only among the RDG patients (*p* = 0.007).

Numerous statistically significant differences were confirmed in the prevalence of functional disorders between the RDG and SHCG (Table 9). The RDG patients were more often mouth breathers (*p* < 0.001) and had atypical swallowing (*p* < 0.001), lingual or labial interposition (*p* < 0.001) and labial incompetence (*p* < 0.001). Only the RDG had patients who had a modified consistency diet (*p* < 0.001). Bruxism (*p* = 0.017) and muscle tone abnormalities (both hypotonia and hypertonia) (*p* < 0.001) were more frequent in the RDG than in the SHCG.

Table 10 details the characteristics of the orthodontic treatment in the RDG and SHCG.

When comparing the two groups, we found statistically significant differences in the percentage of patients who, after undergoing an orthodontic evaluation, ultimately underwent treatment, with fewer RDG patients undergoing treatment after the evaluation (*p* < 0.001). In addition, the desensitization sessions were more necessary in the RDG (*p* = 0.003), particularly those aimed at familiarizing patients with the orthodontic appliances (*p* < 0.001). The RDG patients used exclusively fixed appliances less often and more frequently used mixed appliances (fixed and removable) than the SHCG (*p* = 0.003 and *p* = 0.004, respectively). The mean treatment duration was similar for both groups, except for the therapy with mixed appliances, which was longer for the RDG (*p* < 0.001).

Complications of the orthodontic treatment were more common in the RDG (*p* = 0.015), both in terms of oral (*p* = 0.004) and technical complications (*p* = 0.006). Among the oral complications, gingivitis was more prevalent in the RDG than in the SHCG (*p* = 0.006), and caries and traumatic mucosal ulcers related to the orthodontic appliances were recorded only in the RDG (*p* = 0.013 and *p* = 0.007, respectively). The only technical complication whose prevalence differed between the two groups was recurrent debonding of the device, which was more frequent in the RDG patients (*p* = 0.003). With regard to treatment suspension, when we evaluated the temporary interruption and definitive withdrawal of the appliances, we found no statistically significant differences between the RDG and SHCG.

## 4. Discussion

### 4.1. Anatomical Oral Manifestations in the Rare Disease Patient Group

The most prevalent dental abnormalities in the RDG compared with the SHCG were dental agenesis, microdontia and dental structure abnormalities of the enamel. In the ROMSE database covering a total of 187 RDs, hypodontia was identified as a common finding in 58, oligodontia was identified in 20, and anodontia was identified in 6 [6]. Hypodontia is an especially prevalent dental abnormality in a number of the RDs included in the present series, such as ectodermal dysplasia and Williams syndrome [21]. Untreated moderate–severe hypodontia has a marked negative impact on oral health-related quality of life [22]. At least 12 RDs that progress with microdontia have been identified [6]. A number of the systemic diagnoses of the patients who compose the present series have been related to microdontia, such as Kabuki syndrome [23], Williams syndrome [24], hereditary ectodermal dysplasia [25] and Zellweger syndrome [26]. Dental enamel defects, and especially hypoplasia, have been reported in 30 different RDs [6]. In agreement with other authors, we detected enamel hypoplasia in the present series among the patients with ectodermal dysplasia and Williams syndrome [21] and in a patient with Cri-du-Chat syndrome [27].

The exclusive involvement of the maxilla, overbite and cleft lip/palate were more common in the RDG than in the SHCG. Signs of dysgnathia have been identified in at least 151 RDs, and orofacial clefts have been identified in 148 [6]. As with other authors [8], we detected a considerable number of patients with class II and III malocclusions, open bite, and narrow and high palates (ogival). The ogival palate is a characteristic of a number of the RDs included in the present series, such as Rett syndrome [28], Cri-du-Chat syndrome [27] and Coffin–Siris syndrome [29]. Overbite is a relatively frequent finding in Williams syndrome [30] and Sotos syndrome [31]. Cleft lips/palates were a differential finding between the RDG and SHCG and have been reported in at least 145 RDs [6]. As with previous publications, we detected in the present series clefts in patients diagnosed, among others, with encephalocele [32], Dandy–Walker syndrome [33], CHARGE syndrome [34], Pierre Robin syndrome [35] and Goldenhar syndrome [36].

### 4.2. Oral Functionality

Numerous functional abnormalities were more frequent in the RDG than in the SHCG, including mouth breathing, atypical swallowing, lingual or labial interposition and labial incompetence. Additionally, the RDG had patients who had a modified consistency diet, and bruxism, and muscle tone abnormalities were more frequent. Oromotor deficiencies are a typical finding in more than 40% of patients with RDs [8]. These deficiencies are present in at least 3 of every 4 individuals with some of the diagnoses included in the present series, such as Rett syndrome, Cri-du-Chat syndrome and Williams syndrome [8]. Coinciding with our results, the most common findings included open mouth rest posture (labial incompetence) and orofacial hypotonia [9]. Oromotor deficiencies are closely related to certain morphological disorders and to orofacial dysfunctions such as difficulty swallowing and labial incompetence [9]. Although the orofacial functionality of patients with RDs is generally related to the main target organ/system affected [9], each syndrome can express a specific orofacial dysfunction profile [37]. Malocclusions can have a genetic origin in a number of RDs that affect the orofacial area. In other cases, however, malocclusions can be the result of the respiration pattern, oromotor function, parafunctions, and head position. Evaluating and treating oromotor dysfunction should, therefore, be considered when designing the orthodontic treatment plan [9,38].

### 4.3. Orthodontic Treatment

After undergoing an orthodontic evaluation, only 59.5% of the RDG group underwent treatment. In addition to certain selection criteria previously described in the literature, such as the level of oral hygiene and degree of behavioral control [39,40], two new restrictions were introduced for patients with RDs: (1) retention of the temporary teeth (which has been associated with numerous congenital syndromes) [41,42,43] and (2) refusal by the legal guardians to start the orthodontic treatment when the patient has other severe medical determinants.

In this study, we confirmed that the initial desensitization visits were necessary before starting the orthodontic treatment, as has previously been suggested for patients with intellectual disability [39,44]. Mixed appliances (fixed and removable) were used more frequently in the RDG than in the SHCG. In the patients with congenital syndromes, it is not uncommon to have to resort to orthodontic treatment in two phases, typically due to the complexity of the orthodontic diagnosis [45,46,47]. In overall terms, the treatment duration was similar for the RDG and SHCG, as has previously been reported in children with craniofacial malformations [48] and in patients with special healthcare needs [49]. However, studies have indicated that dental chair sessions are longer than for the general population [48,49], although this information was not collected in our study. The onset of gingivitis and caries is mainly related to deficient oral hygiene. Studies have indicated that when children with special healthcare needs undergo orthodontic treatment, it is particularly difficult to maintain an adequate level of oral hygiene [50,51]. The onset of mouth ulcers in relation to orthodontic appliances is a finding of apparently traumatic origin, whose etiology is still unclear, but has been previously reported in patients with Down syndrome and has been related to dysregulation of the inflammatory response [44,52]. The spontaneous debonding of appliances is one of the most common clinical problems, particularly in fixed orthodontic treatments [53]. Its high prevalence in patients with RDs has not been previously reported, and its cause is unknown, although it could be attributed to the loss of secondary adhesion due to enamel defects, to the presence of parafunctions (e.g., bruxism) and to harmful habits (e.g., inserting objects/fingers into the mouth). A characteristic of orthodontic treatment of the RDG that should be emphasized is that once the treatment had started, the rates of treatment suspension were similar to those of the SHCG.

### 4.4. Limitations

This study has a number of limitations that should be considered when interpreting the results. The present series does not reflect the prevalence of dental-skeletal abnormalities or of orofacial dysfunction in patients with RDs, given that all of the participants were drawn from orthodontic evaluations; for example, the prevalence of malocclusions in the patients with RDs was substantially different from that reported for the general Spanish population [54] but similar to that of the SHCG (healthy but referred for orthodontic evaluation). Given this study’s retrospective nature, the results have a purely descriptive value, given that there is no objective baseline morphological and functional registry, and we, therefore, do not know the severity of the pretreatment findings. Moreover, given the lack of a post-treatment registry, we cannot quantify the results in terms of cosmetics, function, quality-of-life or psychosocial impact.

## 5. Conclusions

Among the patients with RDs seeking orthodontic treatment, a high prevalence of dental-skeletal abnormalities and oromotor dysfunction has been detected, which varies depending on the main target organ/system affected and determines the treatment planning. Approximately 40% of these patients ultimately do not undergo orthodontic treatment due to practitioner criteria, for reasons attributable to the patient or based on the decision of their legal guardians. The orthodontic treatment of patients with RDs has a number of peculiarities such as the need for prior desensitization sessions, longer durations than normal when using mixed appliances (fixed and removable), and the onset of oral and technical complications that can typically be solved without having to discontinue the treatment.

## Figures and Tables

**Table 1:** Distribution of the rare diseases study group.

Disease Category, (*n*)	Diagnosis, (*n*)
Neurological disorders (25)	Basal encephalocele (1)
	SYNGAP1-related developmental and epileptic encephalopathy (1)
	Pontocerebellar hypoplasia (2)
	Hypoplasia/agenesis of the corpus callosum (2)
	Periventricular leukomalacia (3)
	Dandy–Walker malformation (1)
	Arnold–Chiari malformation type 1 (1)
	Microcephaly (3)
	Dravet syndrome (1)
	Endosteal sclerosis-cerebellar hypoplasia syndrome (1)
	Chromosome 15 inversion–duplication syndrome (1)
	Joubert syndrome (1)
	2q23.1 microdeletion syndrome (1)
	Rett syndrome (3)
	Shapiro syndrome (1)
	Tourette syndrome (1)
	West syndrome (1)
Global developmental disorders (12)	Tay–Sachs disease (1)
	Cri-du-Chat syndrome (1)
	De Grouchy syndrome (1)
	Kabuki syndrome (2)
	Xp22.3 microdeletion syndrome (1)
	Prader–Willi syndrome (2)
	Smith–Magenis syndrome (1)
	Williams syndrome (3)
Skeletal dysplasia (10)	Achondroplasia (1)
	Arthrogryposis (1)
	Chondrodysplasia (1)
	Rhizomelic skeletal dysplasia of unknown origin (1)
	Blount disease (1)
	Spina bifida (1)
	Radioulnar terminal transverse meromelia (1)
	Osteogenesis imperfecta (1)
	Myhre syndrome (1)
	Sotos syndrome (1)
Head and neck syndromes (10)	Cherubism (2)
	Apert syndrome (1)
	CHARGE syndrome (2)
	Hallermann–Streiff syndrome (1)
	Pierre Robin syndrome (3)
	Treacher Collins syndrome (1)
Genodermatosis (9)	Ectodermal dysplasia (7)
	Hypomelanosis of Ito (1)
	Gorlin–Goltz syndrome (1)
Sensory disorders (4)	Leber’s congenital amaurosis (1)
	Zellweger spectrum (1)
	Congenital rubella (1)
	Goldenhar syndrome (1)
Intellectual/cognitive impairment (11)	Congenital hypothyroidism (1)
	GRIN 1 mutation (1)
	Alpha thalassemia X-linked intellectual disability syndrome (1)
	Coffin–Siris syndrome (2)
	Chromosome 22q11.21 duplication syndrome (1)
	Smith–Lemli–Opitz syndrome (1)
	Fragile X syndrome (4)
Miscellaneous (13)	Spinal muscular atrophy (1)
	Congenital citrullinemia type 1 (1)
	Glycogenosis type 1b (1)
	Minicore myopathy (1)
	Idiopathic thrombocytopenic purpura (1)
	Alagille syndrome (1)
	Beckwith–Wiedemann syndrome (2)
	Sanfilippo syndrome (1)
	Short bowel syndrome (1)
	1:9 (p31.2, q31) chromosomal translocation (1)
	Chromosome 2 translocation (1)
	Partial trisomy of the long arm of chromosome 16 (1)

**Table 2:** Oral/dental manifestations of the rare diseases patient group, distributed by categories based on target organ/system.

Dental Abnormalities		ND *n* (%)	GDD *n* (%)	SkD *n* (%)	HNS *n* (%)	G *n* (%)	SnD *n* (%)	ICI *n* (%)	M *n* (%)	Total *n* (%)
**Tooth Eruption**	Chronology	1	0	1	3	0	1	2	0	8
		(4.0)	(0)	(10.0)	(30.0)	(0)	(25.0)	(18.1)	(0)	(8.5)
	Ectopia	1	2	4	6	1	0	1	3	18
		(4.0)	(16.7)	(40.0)	(60.0)	(11.1)	(0)	(9.0)	(23.1)	(19.1)
**Number**	Agenesis	2	2	4	3	9	2	0	1	23
		(8.0)	(16.7)	(40.0)	(30.0)	(100)	(50.0)	(0)	(7.7)	(24.5)
	Supernumerary	2	0	0	0	1	1	1	0	5
		(8.0)	(0)	(0)	(0)	(11.1)	(25.0)	(9.0)	(0)	(5.3)
**Morphology**	Size	0	0	0	0	4	1	1	0	6
		(0)	(0)	(0)	(0)	(44.4)	(25.0)	(9.0)	(0)	(6.4)
	Appearance	0	0	1	1	4	2	0	0	8
		(0)	(0)	(10.0)	(10.0)	(44.4)	(50.0)	(0)	(0)	(8.5)
**Structure**	Enamel	0	2	0	3	1	1	2	2	11
		(0)	(16.7)	(0)	(30.0)	(11.1)	(25.0)	(18.1)	(15.4)	(11.7)
	Dentin	0	0	1	0	0	0	0	0	1
		(0)	(0)	(10)	(0)	(0)	(0)	(0)	(0)	(1.1)

ND, neurological disorders; GDD, global developmental disorders; SkD, skeletal dysplasia; HNS, head and neck syndromes; G, genodermatosis; SnD, sensory disorders; ICI, intellectual/cognitive impairment; M, miscellaneous.

**Table 3:** Orthodontic oral manifestations of the rare diseases patient group, distributed by categories according to target organ/system.

Orthodontic Variables		ND *n* (%)	GDD *n* (%)	SkD *n* (%)	HNS *n* (%)	G *n* (%)	SnD *n* (%)	ICI *n* (%)	M *n* (%)	Total *n* (%)
**Angle class**	I	5	1	0	1	5	1	1	4	18
		(20.0)	(8.3)	(0)	(10.0)	(55.6)	(25.0)	(9.1)	(30.8)	(19.1)
	II	15	5	4	5	0	0	6	2	37
		(60.0)	(41.7)	(40.0)	(50.0)	(0)	(0)	(54.5)	(15.4)	(39.4)
	III	5	6	6	4	4	3	4	7	39
		(20.0)	(50.0)	(60.0)	(40.0)	(44.4)	(75.0)	(36.4)	(53.8)	(41.5)
**Bone base affected**	Maxillary	4	4	3	2	4	1	2	3	23
		(16.0)	(33.3)	(30.0)	(20.0)	(44.4)	(25.0)	(18.2)	(23.1)	(24.5)
	Mandibular	7	4	2	2	0	0	3	3	21
		(28.0)	(33.3)	(20.0)	(20.0)	(0)	(0)	(27.3)	(23.1)	(22.3)
	Mixed	14	4	5	6	5	3	6	7	50
		(56.0)	(33.3)	(50.0)	(60.0)	(55.6)	(75.0)	(54.5)	(53.8)	(53.2)
**Biotype**	Mesofacial	10	7	7	7	9	2	9	5	56
		(40.0)	(58.3)	(70.0)	(70.0)	(100)	(50.0)	(81.8)	(38.5)	(59.6)
	Brachyfacial	3	1	0	0	0	0	0	0	4
		(12.0)	(8.3)	(0)	(0)	(0)	(0)	(0)	(0)	(4.3)
	Dolichofacial	12	4	3	3	0	2	2	8	34
		(48.0)	(33.3)	(30.0)	(30.0)	(0)	(50.0)	(18.2)	(61.5)	(36.2)
**Vertical**	Open bite	7	3	3	2	0	2	3	6	26
		(28.0)	(25.0)	(30.0)	(20.0)	(0)	(50.0)	(27.3)	(46.2)	(27.7)
	Overbite	6	1	2	1	0	0	0	0	10
		(24.0)	(8.3)	(20.0)	(10.0)	(0)	(0)	(0)	(0)	(10.6)
**Crossbite**	Unilateral	3	0	1	0	0	1	1	2	8
		(12.0)	(0)	(10.0)	(0)	(0)	(25.0)	(9.1)	(15.4)	(8.5)
	Bilateral	3	2	2	4	1	2	3	3	20
		(12.0)	(16.7)	(20.0)	(40.0)	(11.1)	(50.0)	(27.3)	(23.1)	(21.3)
**Scissor bite**		0	1	0	1	0	0	0	0	2
		(0)	(8.3	(0)	(10	(0)	(0)	(0)	(0)	(2.1)
**Maxillary compression**		8	4	6	7	2	3	6	5	41
		(32.0)	(33.3)	(60.0)	(70.0)	(22.2)	(75.0)	(54.5)	(38.5)	(43.6)
**Cleft lip/palate**		2	0	0	2	0	2	0	1	7
		(8.0)	(0)	(0)	(20.0)	(0)	(50.0)	(0)	(7.7)	(7.4)

ND, neurological disorders; GDD, global developmental disorders; SkD, skeletal dysplasia; HNS, head and neck syndromes; G, genodermatosis; SnD, sensory disorders; ICI, intellectual/cognitive impairment; M, miscellaneous.

**Table 4:** Oral functionality of the rare diseases patient group, distributed by categories according to target organ/system.

Functional Variables		ND *n* (%)	GDD *n* (%)	SkD *n* (%)	HNS *n* (%)	G *n* (%)	SnD *n* (%)	ICI *n* (%)	M *n* (%)	Total *n* (%)
**Mouth breathing**		11	6	3	8	0	3	7	10	48
		(44.0)	(50.0)	(30.0)	(80.0)	(0)	(75.0)	(63.6)	(76.9)	(51.1)
**Atypical swallowing**		6	4	2	3	0	1	5	5	26
		(24.0)	(33.3)	(20.0)	(30.0)	(0)	(25.0)	(45.4)	(38.5)	(27.7)
**Interposition**	Lingual	6	6	3	3	0	1	5	5	29
		(24.0)	(50.0)	(30.0)	(30.0)	(0)	(25.0)	(45.5)	(38.5)	(30.9)
	Lower lip	1	2	0	1	0	0	3	1	8
		(4.0)	(16.7)	(0)	(10.0)	(0)	(0)	(27.3)	(7.7)	(8.5)
**Frenulum disorder**	Upper	1	0	0	0	0	0	0	0	1
		(4.0)	(0)	(0)	(0)	(0)	(0)	(0)	(0)	(1.1)
	Lower	1	0	1	1	0	0	0	0	3
		(4.0)	(0)	(10.0)	(10.0)	(0)	(0)	(0)	(0)	(3.1)
**Labial incompetence**		11	7	3	5	0	3	6	10	45
		(44.0)	(58.3)	(30.0)	(50.0)	(0)	(75.0)	(54.5)	(76.9)	(47.9)
**Feeding**	Oral	24	12	10	9	9	4	11	12	91
		(96.0)	(100)	(100)	(90.0)	(100)	(100)	(100)	(92.3)	(96.8)
	Modified consistency	4	1	0	2	0	2	2	1	12
		(16.0)	(8.3)	(0)	(20.0)	(0)	(50.0)	(18.2)	(7.7)	(12.8)
	Non-oral	1	0	0	1	0	0	0	1	3
		(4.0)	(0)	(0)	(10.0)	(0)	(0)	(0)	(7.7)	(3.2)
**Parafunction**	Bruxism	9	3	4	2	0	1	4	2	25
		(36)	(25)	(40)	(20)	(0)	(25)	(36.4)	(15.4)	(26.6)
	Chews hands or objects	3	0	0	0	0	0	1	0	4
		(12)	(0)	(0)	(0)	(0)	(0)	(9.1)	(0)	(4.3)
**Muscle tone**	Hypotonia	12	5	1	3	0	0	4	4	29
		(48.0)	(41.7)	(10.0)	(30.0)	(0)	(0)	(36.4)	(30.8)	(30.9)
	Hypertonia	3	4	5	3	0	4	1	2	22
		(12.0)	(33.3)	(50.0)	(30.0)	(0)	(100)	(9.1)	(15.4)	(23.4)

ND, neurological disorders; GDD, global developmental disorders; SkD, skeletal dysplasia; HNS, head and neck syndromes; G, genodermatosis; SnD, sensory disorders; ICI, intellectual/cognitive impairment; M, miscellaneous.

**Table 5:** Reasons for not performing the orthodontic treatment in the rare diseases patient group, distributed by categories according to target organ/system.

		ND *n* (%)	GDD *n* (%)	SkD *n* (%)	HNS *n* (%)	G *n* (%)	SnD *n* (%)	ICI *n* (%)	M *n* (%)	Total *n* (%)
Patients who did not start the treatment *		11	6	4	3	1	1	6	6	38
		(44.0)	(50.0)	(40.0)	(30.0)	(11.1)	(25)	(54.5)	(46.2)	(40.4)
**Reasons attributable to the patient**	Any reason	9	6	1	2	0	1	3	4	26
		(81.8)	(100)	(25.0)	(66.6)	(0)	(100)	(50.0)	(66.6)	(68.4)
	Uncontrolled disease	0	0	0	1	0	0	0	2	3
		(0)	(0)	(0)	(33.3)	(0)	(0)	(0)	(33.3)	(7.8)
	Pharmacotherapy	0	0	0	0	0	0	0	1	1
		(0)	(0)	(0)	(0)	(0)	(0)	(0)	(16.6)	(2.6)
	Difficulty breathing	0	0	0	0	0	0	0	0	0
		(0)	(0)	(0)	(0)	(0)	(0)	(0)	(0)	(0)
	Lack of collaboration	9	5	1	2	0	1	2	1	21
		(81.8)	(83.3)	(25.0)	(66.6)	(0)	(100)	(33.3)	(16.6)	(55.2)
	Small mouth opening	0	0	0	0	0	1	1	0	2
		(0)	(0)	(0)	(0)	(0)	(100)	(16.6)	(0)	(5.2)
	Difficulty eating	1	0	0	2	0	0	0	0	3
		(9.0)	(0)	(0)	(66.6)	(0)	(0)	(0)	(0)	(7.8)
	Distress talking	0	0	0	0	0	1	1	0	2
		(0)	(0)	(0)	(0)	(0)	(100)	(16.6)	(0)	(5.2)
	Exacerbated nausea	0	0	0	0	0	0	1	0	1
		(0)	(0)	(0)	(0)	(0)	(0)	(16.6)	(0)	(2.6)
	Deficient oral hygiene	3	4	0	1	0	1	1	4	14
		(27.2)	(66.6)	(0)	(33.3)	(0)	(100)	(16.6)	(66.6)	(36.8)
	Caries	0	0	0	1	0	1	0	0	2
		(0)	(0)	(0)	(33.3)	(0)	(100)	(0)	(0)	(5.2)
**Decision of the guardians**		6	2	3	1	1	0	5	4	22
		(54.5)	(33.3)	(75.0)	(33.3)	(100)	(0)	(83.3)	(66.6)	(57.8)
**Practitioner’s opinion**	Any opinion	7	4	2	2	1	1	4	6	27
		(63.6)	(66.6)	(50.0)	(66.6)	(100)	(100)	(66.6)	(100)	(71.0)
	Wait for bone growth	1	2	1	1	1	1	3	3	13
		(9.0)	(33.3)	(25.0)	(33.3)	(100)	(100)	(50.0)	(50.0)	(34.2)
	Wait for tooth replacement	6	4	2	2	1	1	4	6	26
		(54.5)	(66.6)	(50.0)	(66.6)	(100)	(100)	(66.5)	(100)	(68.4)
	Surgical indication	0	0	1	1	1	0	0	0	3
		(0)	(0)	(25.0)	(33.3)	(100)	(0)	(0)	(0)	(7.8)

The percentage (%) signs are with respect to the number of individuals in each category of the RDG without orthodontic treatment; * % with respect to the number of individuals in each category of the RDG. ND, neurological disorders; GDD, global developmental disorders; SkD, skeletal dysplasia; HNS, head and neck syndromes; G, genodermatosis; SnD, sensory disorders; ICI, intellectual/cognitive impairment; M, miscellaneous.

**Table 6:** Characteristics of the orthodontic treatment in the rare diseases patient group, distributed by categories according to target organ/system.

		ND *n* (%)	GDD *n* (%)	SkD *n* (%)	HNS *n* (%)	G *n* (%)	SnD *n* (%)	ICI *n* (%)	M *n* (%)	Total *n* (%)
Patients with treatment *		14	6	6	7	8	3	5	7	56
		(56.0)	(50.0)	(60.0)	(70.0)	(88.8)	(75.0)	(45.5)	(53.9)	(59.5)
**Prior desensitization**		7	2	2	3	0	1	1	1	17
		(50.0)	(33.3)	(33.3)	(42.8)	(0)	(33.3)	(20.0)	(14.2)	(30.3)
**Type of device**	Removable	8	4	3	3	0	1	3	3	25
		(57.1)	(66.6)	(50)	(42.8)	(0)	(33.3)	(60.0)	(42.8)	(44.6)
	Fixed	10	6	4	7	8	3	4	7	49
		(71.7)	(100)	(66.6)	(100)	(100)	(100)	(80.0)	(100)	(87.5)
	Removable and Fixed	4	4	1	3	0	1	2	3	18
		(28.5)	(66.6)	(16.6)	(42.8)	(0)	(33.3)	(40.0)	(42.8)	(32.1)
**TxD [range]**	Removable	22.3	19.2	22.3	18.3	0	10.0	28.0	22.0	20.3
		[9–36]	[8–26]	[9–40]	[12–24]	[0]	[10–10]	[12–48]	[18–24]	[8–48]
	Fixed	23.5	23.6	38.7	24.8	45.7	54	35.5	31.4	34.6
		[3–38]	[9–32]	[17–60]	[4–44]	[6–74]	[24–78]	[8–50]	[22–40]	[3–78]
	Removable and Fixed	27.9	36.5	37.0	32.7	45.7	57.3	45.2	40.8	39.6
		[3–72]	[26–49]	[37–37]	[16–57]	[6–74]	[57–57]	[32–62]	[22–60]	[3–88]
**Onset of complication**		9	3	6	6	6	2	4	5	41
		(64.2)	(50.0)	(100)	(85.7)	(75.0)	(66.6)	(80.0)	(71.4)	(73.2)
**Oral complications**	Any complication	5	3	4	6	5	1	3	3	30
		(35.7)	(50.0)	(66.6)	(85.7)	(62.5)	(33.3)	(60.0)	(42.8)	(53.5)
	Gingivitis	5	3	3	6	5	1	3	3	29
		(35.7)	(50.0)	(50)	(85.7)	(62.5)	(33.3)	(60.0)	(42.8)	(51.7)
	Caries	0	1	1	3	0	0	1	0	6
		(0)	(16.6)	(16.6)	(42.8)	(0)	(0)	(20.0)	(0)	(10.7)
	Rhizolysis	0	1	1	0	0	0	0	0	2
		(0)	(16.6)	(16.6	(0)	(0)	(0)	(0)	(0)	(3.5)
	Ulcer	1	0	1	5	0	0	0	0	7
		(7.1)	(0)	(16.6)	(71.4)	(0)	(0)	(0)	(0)	(12.5)
**Systemic complications**	Uncontrolled systemic disease	2	0	0	1	0	0	0	0	3
		(14.2)	(0)	(0)	(14.2)	(0)	(0)	(0)	(0)	(5.3)
**Technical complications**	Any complication	7	2	6	6	4	1	4	5	35
		(50.0)	(33.3)	(100)	(85.7)	(50.0)	(33.3)	(80.0)	(71.4)	(62.5)
	Displaced arch	0	0	0	2	4	1	1	1	9
		(0)	(0)	(0)	(28.5)	(50.0)	(33.3)	(20.0)	(14.2)	(16.0)
	Recurrent debonding	6	2	4	6	3	1	3	4	29
		(42.8)	(33.3)	(66.6)	(85.7)	(37,5)	(33.3)	(60.0)	(57.1)	(51.7)
	Seldom used	1	0	3	3	0	0	1	2	10
		(7.1)	(0)	(50.0)	(42.8)	(0)	(0)	(20.0)	(28.5)	(17.8)
	Breakage	5	0	0	1	1	0	0	1	8
		(35.7)	(0)	(0)	(14.2)	(12.5)	(0)	(0)	(14.2)	(14.2)
	Loss	0	0	0	0	0	0	0	0	0
		(0)	(0)	(0)	(0)	(0)	(0)	(0)	(0)	(0)
**Treatment stopped**	Temporary	5	2	1	4	0	0	0	0	12
		(35.7)	(33.3)	(16.6)	(57.1)	(0)	(0)	(0)	(0)	(21.4)
	Definitive	0	0	2	0	2	0	0	0	4
		(0)	(0)	(33.3)	(0)	(25.0)	(0)	(0)	(0)	(7.1)

The percentage signs are with respect to the number of individuals in each category of the RDG with orthodontic treatment; * % with respect to the number of individuals in each category of the RDG. ND, neurological disorders; GDD, global developmental disorders; SkD, skeletal dysplasia; HNS, head and neck syndromes; G, genodermatosis; SnD, sensory disorders; ICI, intellectual/cognitive impairment; M, miscellaneous; TxD, mean treatment duration (in months).

**Table 7:** Oral/dental manifestations of the rare diseases patient group (RDG) and systemically healthy control group (SHCG).

Dental Abnormalities		RDG *n* (%)	SHCG *n* (%)	*p*
**Tooth Eruption**	Chronology	8 (8.5)	8 (8.5)	1.000 (1)
	Ectopia	18 (19.1)	19 (20.2)	0.854 (1)
**Number**	Agenesis	23 (24.5)	4 (4.3)	<0.001 (1)
	Supernumerary	5 (5.3)	0 (0)	-
**Morphology**	Size	6 (6.4)	5 (5.4)	0.027 (2)
	Appearance	8 (8.5)	4 (4.3)	0.233 (1)
**Structure**	Enamel	11 (11.7)	2 (2.1)	0.010 (2)
	Dentin	1 (1.1)	0 (0)	

(1) chi-squared test; (2) Fisher’s exact test.

**Table 8:** Orthodontic manifestations of the rare diseases patient group (RDG) and systemicallyhealthy control group (SHCG).

Orthodontic Variables		RDG *n* (%)	SHCG *n* (%)	*p*
**Angle class**	I	18 (19.1)	30 (31.9)	0.060 (1)
	II	37 (39.4)	38 (40.4)	
	III	39 (41.5)	26 (27.7)	
**Bone base affected**	Maxillary	23 (24.5)	6 (6.4)	0.002 (1)
	Mandibular	21 (22.3)	22 (23.4)	
	Mixed	50 (53.2)	66 (70.2)	
**Biotype**	Mesofacial	56 (59.6)	62 (66)	0.114 (1)
	Brachyfacial	4 (4.3)	9 (9.6)	
	Dolichofacial	34 (36.2)	23 (24.5)	
**Vertical**	Open bite	26 (27.7)	20 (21.3)	0.309 (1)
	Overbite	10 (10.6)	6 (6.4)	0.013 (1)
**Crossbite**	Unilateral	8 (8.5)	17 (18.1)	0.073 (1)
	Bilateral	20 (21.3)	12 (12.8)	
**Scissor bite**		2 (2.1)	4 (4.3)	0.682 (2)
**Maxillary compression**		41 (43.6)	52 (55.3)	0.109 (1)
**Cleft lip/palate**		7 (7.4)	0 (0)	0.007 (1)

(1) chi-squared test; (2) Fisher’s exact test.

**Table 9:** Oral functionality of the rare diseases patient group (RDG) and systemically healthy control group (SHCG).

Functional Variables		RDG *n* (%)	SHCG *n* (%)	*p*
**Mouth breathing**		48 (51.1)	24 (25.5)	<0.001 (1)
**Atypical swallowing**		26 (27.7)	2 (2.1)	<0.001 (1)
**Tissue interposition**	Lingual	29 (30.9)	2 (2.1)	<0.001 (1)
	Lower lip	8 (8.5)	0 (0)	
**Frenulum disorder**	Upper	1 (1)	2 (2.1)	0.621 (2)
	Lower	3 (3.1)	0 (0)	
**Labial incompetence**		45 (47.9)	13 (13.8)	<0.001 (1)
**Feeding**	Modified consistency	12 (12.8)	0 (0)	<0.001 (1)
	Non-oral	3 (3.2)	0 (0)	0.246 (2)
**Parafunctions**	Bruxism	25 (26.6)	12 (12.8)	0.017 (1)
	Chews hands or objects	4 (4.3)	0 (0)	0.121 (1)
**Muscle tone**	Hypotonia	29 (30.9)	10 (10.6)	<0.001 (1)
	Hypertonia	22 (23.4)	9 (9.6)	

(1) chi-squared test; (2) Fisher’s exact test.

**Table 10:** Characteristics of the orthodontic treatment in the rare diseases patient group (RDG) and systemically healthy control group (SHCG).

Variables of Orthodontic Treatment		RDG *n* (%)	SHCG *n* (%)	*p*
**Individuals who started orthodontic treatment**		56 (59.2)	88 (93.6)	<0.001 (1)
**Need for prior desensitization**	Desensitization	17 (18.1)	4 (4.3)	0.003 (1)
	General dental desensitization	17 (18.1)	4 (4.3)	0.003 (1)
	Orthodontic desensitization	17 (18.1)	0 (0)	<0.001 (1)
**Type of appliances**	Removable	25 (26.6)	24 (25.5)	0.868 (1)
	Fixed	49 (52.1)	69 (73.4)	0.003 (1)
	Both	18 (19.1)	5 (5.3)	0.004 (1)
**Mean treatment duration, months [range]**	Removable	21.5 [8–48]	18.9 [1–26]	0.318 (3)
	Fixed	32.3 [3–78]	28.2 [6–48]	0.450 (3)
	Both	37.9 [3–88]	27.2 [15–48]	<0.001 (3)
**Onset of complications**		42 (44.7)	26 (27.7)	0.015 (1)
**Onset of oral complications**	≥1	31 (33)	14 (14.9)	0.004 (1)
	Gingivitis	30 (31.9)	14 (14.9)	0.006 (1)
	Caries	6 (6.4)	0 (0)	0.013 (1)
	Radicular resorption	2 (2.1)	0 (0)	0.497 (2)
	Ulcers	7 (7.4)	0 (0)	0.007 (1)
**Onset of systemic complications**	Uncontrolled systemic disease	3 (3.2)	-	0.246 (2)
**Onset of technical complications**	≥1	35 (37.2)	18 (19.1)	0.006 (1)
	Displaced arch	9 (9.6)	4 (4.3)	0.151 (1)
	Recurrent debonding	29 (30.9)	12 (12.8)	0.003 (1)
	Seldom used	10 (10.6)	8 (8.5)	0.620 (1)
	Breakage	8 (8.5)	4 (4.3)	0.233 (1)
	Loss	0 (0)	0 (0)	-
**Treatment suspension**	Temporary	13 (13.8)	8 (8.5)	0.056 (2)
	Definitive	4 (4.3)	0 (0)	

The percentages are with respect to the number of individuals in each group; ≥1, some complication; (1) chi-squared test; (2) Fisher’s exact test; (3) Wilcoxon rank-sum test with continuity correction.

## Data Availability

The data that support the findings of this study are available on request from the corresponding authors.

## Supplementary Materials

The following supporting information can be downloaded at: https://www.mdpi.com/article/10.3390/jcm11061527/s1. Figure S1: Osteogenesis imperfecta, Figure S2: Kabuki syndrome, Figure S3: Goldenhar syndrome, Figure S4: Ectodermal dysplasia.
