# Supplementary material for: Rare Disorders: Diagnosis and Therapeutic Planning for Patients Seeking Orthodontic Treatment
Source: J Clin Med. 2022 Mar 10;11(6):1527. doi: 10.3390/jcm11061527 (PMC8954363; doi:10.3390/jcm11061527)
Supplement: Supplementary file 1 [file jcm-11-01527-s001.zip › jcm-1594917-supplementary.pdf]

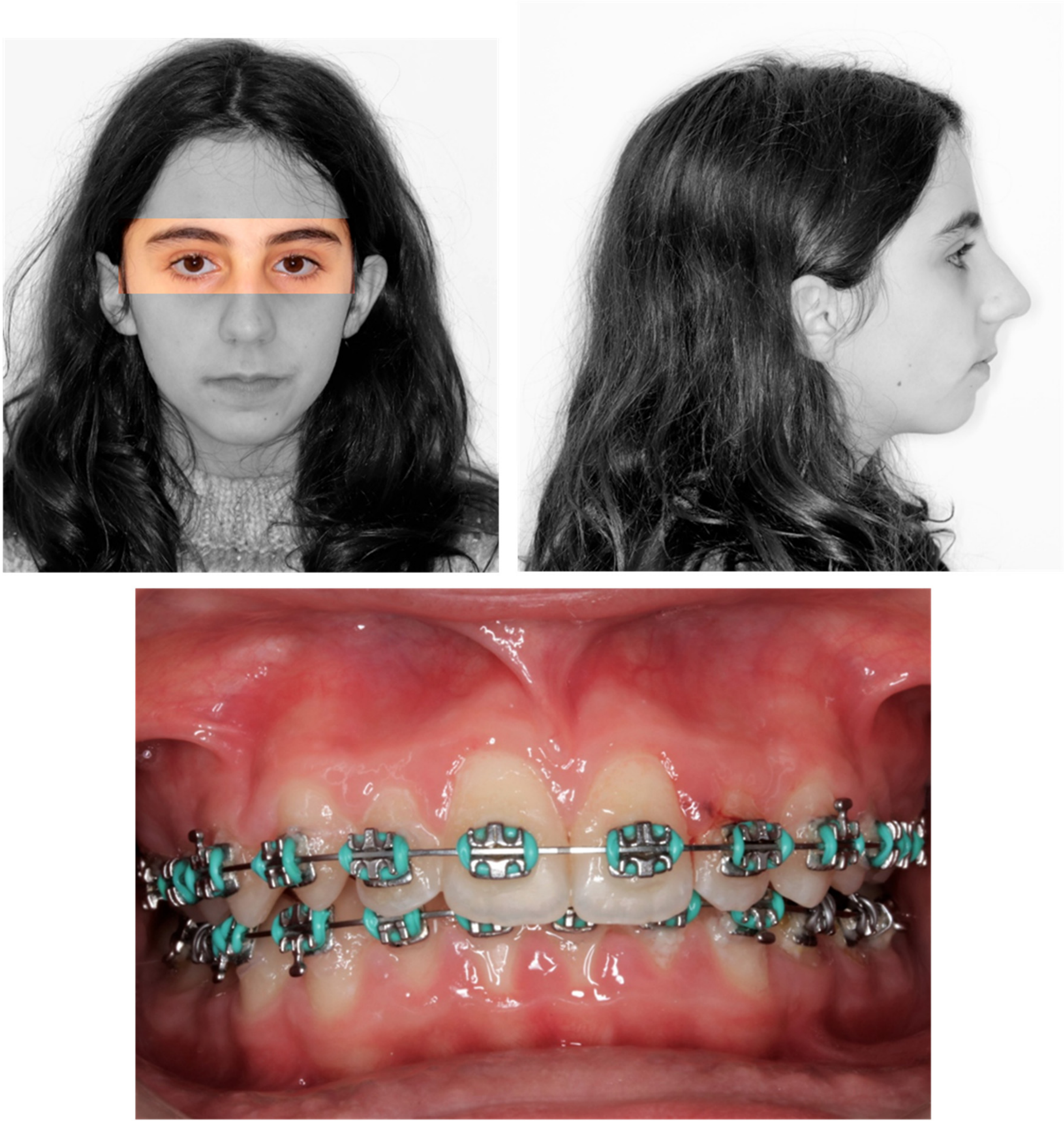

Figure S1. Osteogenesis imperfecta.

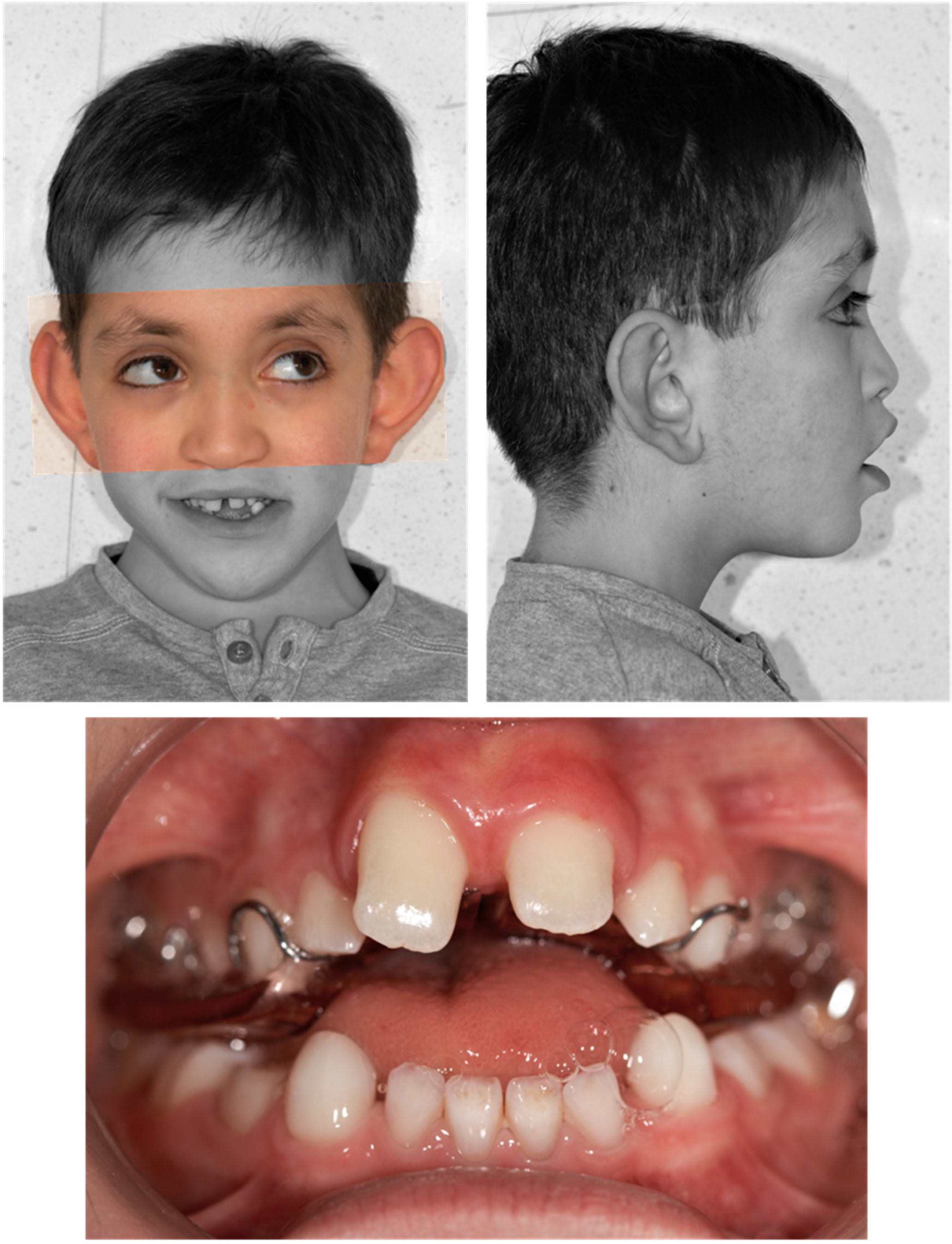

Figure S2. Kabuki syndrome.

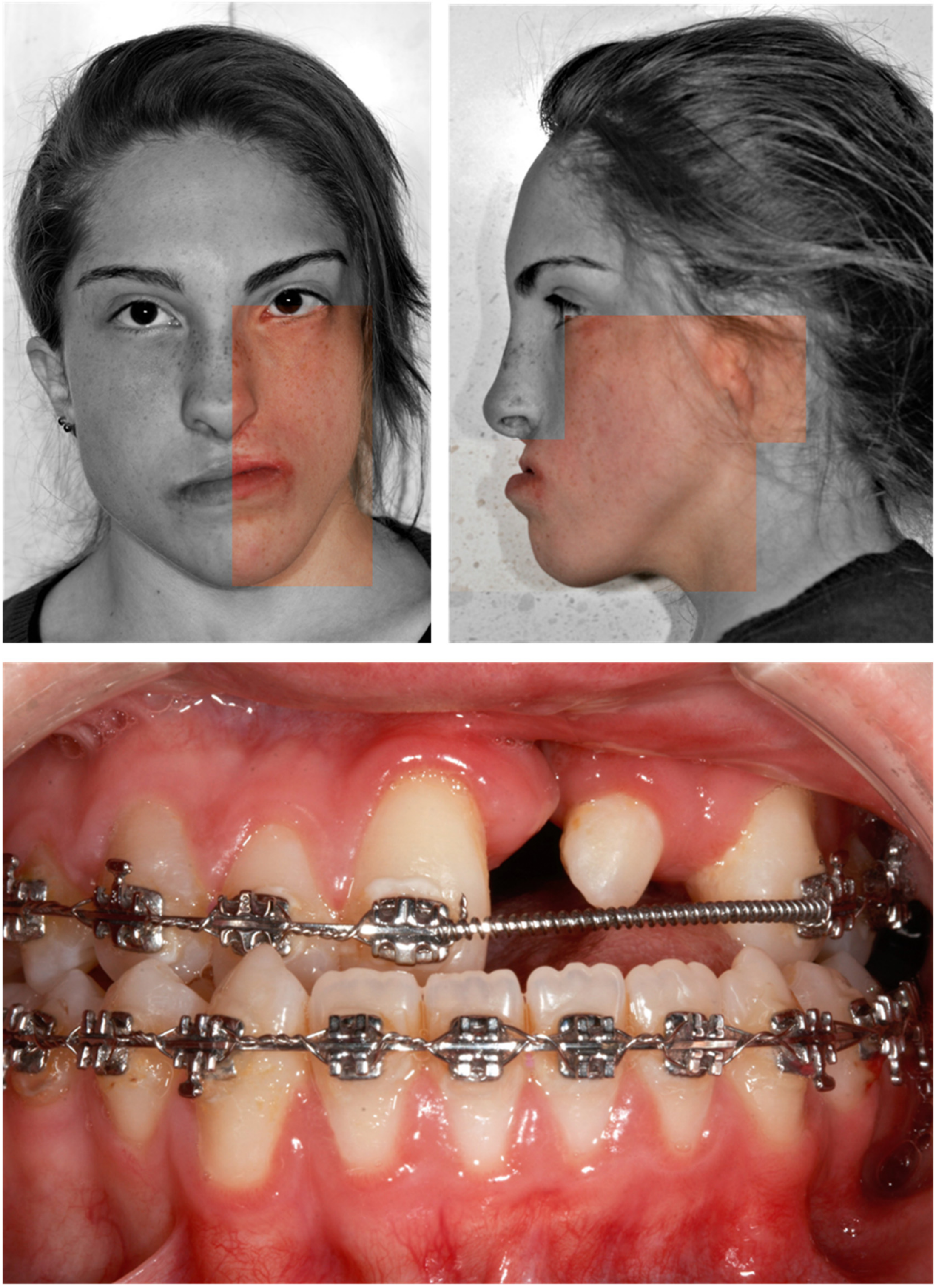

Figure S3. Goldenhar syndrome.

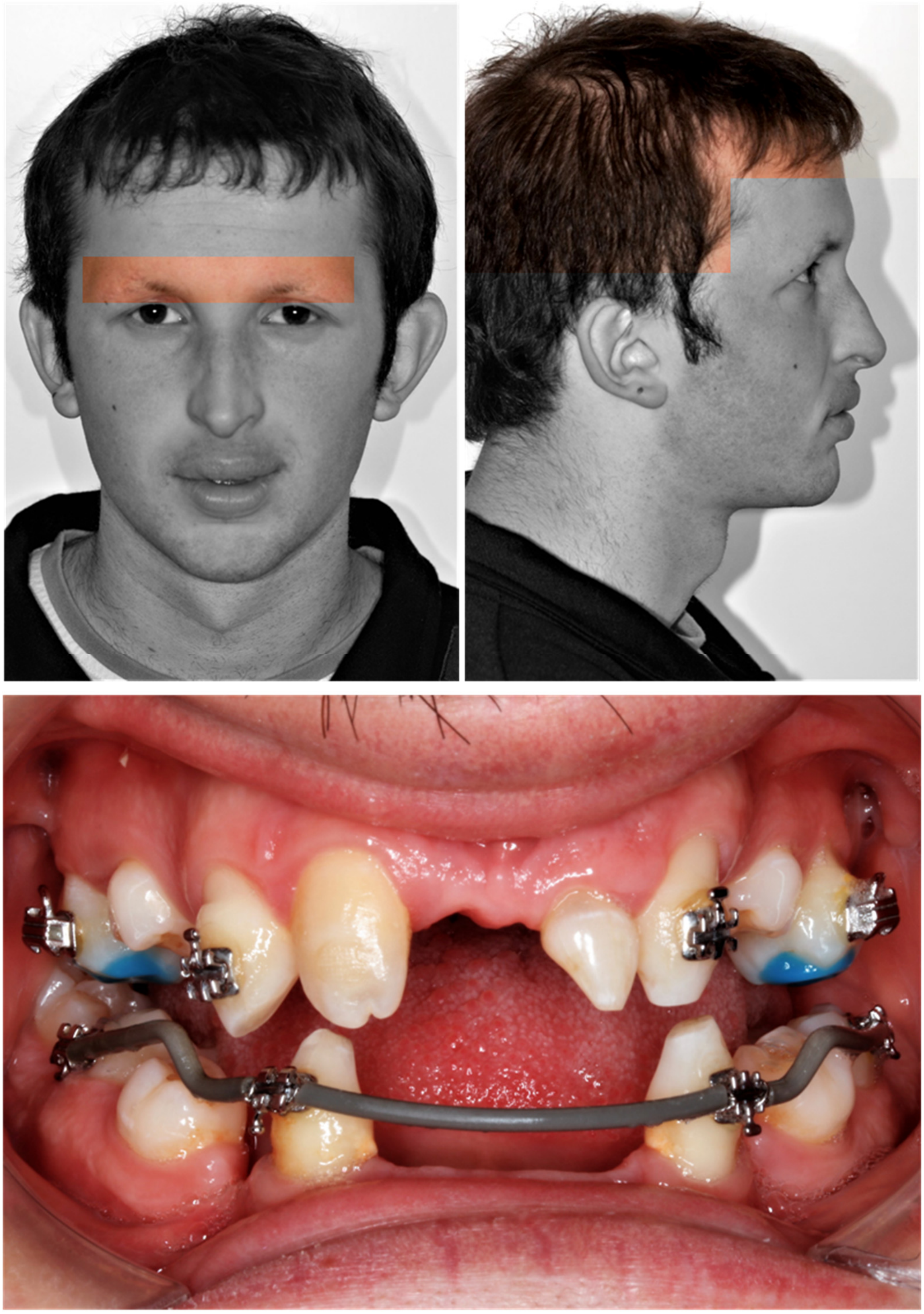

Figure S4. Ectodermal dysplasia.
